# Investigation of the Association between 5-Hydroxytryptamine Transporter Gene-Linked Polymorphic Region with Type 2 Diabetes Mellitus, Obesity and Biochemical Profiles of Serum in Iranian Population

**Published:** 2019

**Authors:** Azizeh Asadzadeh, Hooria Seyedhosseini Ghaheh, Fatemeh Sholehvar, Mohammadali Takhshid, Mohammad Mehdi Naghizadeh

**Affiliations:** 1. Department of Biology, University of Nourdanesh Institute of Higher Education, Meymeh, Isfahan, Iran; 2. Young Researchers and Elites Club, Science and Research Branch, Islamic Azad University, Tehran, Iran; 3. Department of Biology, Faculty of Science, Zand Institute of Higher Education, Shiraz, Iran; 4. Diagnostic Laboratory Sciences and Technology Research Center, School of Paramedical Sciences, Shiraz University of Medical Sciences, Shiraz, Iran; 5. Noncommunicable Diseases Research Center, Fasa University of Medical Sciences, Fasa, Iran

**Keywords:** *5-HTT* gene, 5-HTTLPR polymorphism, Serotonin, Type 2 diabetes mellitus

## Abstract

**Background:**

Type 2 Diabetes Mellitus (T2DM) is a serious problem in the world. 5-Hydroxytryptamine (5-HT, serotonin) plays an important role in obesity, glucose control and insulin resistance. The polymorphism of the serotonin transporter gene linked promoter region (5-HTTLPR) might influence 5-HTT expression and serotonin uptake. The polymorphism results in two alleles of L (Long) and S (Short). The aim of the present study was to evaluate the association between 5-HTTLPR genotypes in type 2 diabetes mellitus (T2DM), obesity as well as serum biochemical profiles in Iranian population from 2012 until 2015.

**Methods:**

180 patients with T2DM and 180 controls were selected and the frequency of S and L alleles was determined by PCR. Then, the relationship between genotypes, body mass index (BMI) and serum biochemical variables was investigated.

**Results:**

The frequency of S and L alleles in experimental and control groups was the same [for the L allele p=0.754, OR (95%CI)=1.103 (0.597 to 2.041) and for the S allele p=0.906, OR (95%CI)=(0.490 to 1.676)]. However, the mean triglyceride, cholesterol, LDL-C, systolic and diastolic blood pressure levels in the diabetic subjects with LL genotype were significantly higher than LS and SS genotypes (p<0.001) in this population.

**Conclusion:**

The L allele of 5-HTTLPR was related to the increased serum lipids and blood pressure in the diabetic patients. However, there was no relationship between the polymorphism of 5-HTTLPR L/S and T2DM in Iranian population.

## Introduction

Serotonin (5-hydroxytryptamine or 5-HT) synthesis occurs in the periphery within the gut neurons and enterochromaffin cells and centrally within the neurons of the raphe in the brain stem 1,2. Some functional effects of 5-HT include sleep regulation, mood control, voiding, circadian functions, controlling body temperature, body weight, feeding behavior, intestinal motility, and urine storage 3–5. One of the most important regulators for body energy balancing is serotonin 6. Serotonin also controls the glucose homeostasis out of the nervous system 7. However, 5-HTC2 receptor agonists increase the glucose tolerance and improve T2DM 8.

The serotonin transporter (SERT; 5-Hydroxytryptamin transporter; 5-HTT) is one of the important parts of serotonergic body system which is responsible for the reuptake of 5-HT from the synaptic cleft and regulates serotonin signaling in the brain 9. This transporter is in the membrane of neurons, intestine enterochromaffin cells, and platelets and controls the intensity and the time effect of serotonin by transporting serotonin from the synaptic space into the cells 10,11.

The human *5-HTT* gene is located on chromosome 17 at 17q11.2-17q11.12 3. The 5-HTTLPR is a polymorphic region in the promoter of this gene which consists of a 44 base pair fragment with different repetitions and leads to short allele (S) and long allele (L). The S allele and L allele have respectively 14 and 16 repetitive sequences in the polymorphic promoter region 12. 5-HTTLPR polymorphism affects both the transcription and *5-HTT* gene expression. The transcription level of S allele is 2 to 3 times lower than L allele. Therefore, the cells carrying S allele have a lower number of 5-HTT as well as lower efficacy for re-absorption of serotonin 13,14.

Elimination of 5-HTC2 receptor gene in experimental mice led to insulin resistance and T2DM 15,16. Also, several studies investigated genetic association of serotonin transporter gene (SLC6A4), 5-HTTLPR polymorphism, with Type 2 Diabetes Mellitus (T2DM) 6,17–19 and the relation between the SLC6A4 alleles with obesity 20,21. In this study, the association of 5-HTT LPR polymorphism with T2DM, Body Mass Index (BMI) (as a measure of obesity), and biochemical profiles of serum in Iranian population was investigated in order to be able to treat diabetic patients in future. But since the relation between T2DM and 5-HTTLPR polymorphism was not the same in different populations, so it is necessary to investigate these relations in each country separately.

Our study suffered from some limitations, such as a relatively small sample size and a lack of data on the lifestyle habits of patients (*e.g*., active smoking, quality of sleep, and diet).

## Materials and Methods

This study was carried out to evaluate the association of 5-HTTLPR with T2DM, obesity and biochemical profiles of serum in Iranian population during 2012–15. The clinical experiments were performed in Shiraz University of Medical Sciences (Shiraz, Iran).

### Study population

A total of 180 patients with T2DM and 180 non-diabetic healthy control subjects (84 males and 96 females in each group) were randomly recruited from Iranian general population. Iranian patients with T2DM were recruited from Diabetes and Metabolic Disorders Specialty Clinic (Tehran) who came from all over Iran and were referred to that clinic from 2012 to 2015. The diagnosis of T2DM was based on the guidelines of WHO 22. A specially-designed checklist was used to gather the information about the epidemiologic indicators, such as location, age, sex, employment, and time of being infected by diabetes. The questionnaire was completed by the patients.

The members of the control group were randomly selected from sex and age matched healthy individuals who had no symptoms of T2DM who were referred to the blood transfusion organizations from approximately all over Iran. Written informed consent was obtained from the all the subjects. In addition, the study was approved by the Ethics Committee of Shiraz University of Medical Sciences, Shiraz, Iran.

### Clinical measures

A five *ml* blood sample was obtained from each subject after an overnight fasting. The serum samples were isolated and some of biochemical parameters such as serum cholesterol, Triglyceride (TG), Fasting Blood Glucose (FBG) and glycosylated hemoglobin (HbA1C) were assessed by auto analyzer (BIOLIS50i Superior, Tokyo, Japan). BMI was calculated as the ratio of weight to the square of the height (*kg/m*^2^) 17*.* The blood pressure measurement was determined while the subjects were in the sitting position. Blood pressure was taken by using a periodically calibrated mercury sphygmomanometer.

### Genotyping

Genomic DNA was extracted from white blood cells for each patient and healthy individual by using Qia Amp DNA Mini Kit (Qiagen Inc, Valencia, CA, USA). Then, the polymorphic promoter region was proliferated by polymerase chain reaction (PCR) as previously described by Nazem *et al* 19 to determine 5-HTTLPR di-allelic polymorphism. The PCR reaction was performed in the total volume of 20 *µl* containing 100 *ng* DNA, 2.0 *mM* MgCl_2_, 0.2 *mM* dNTPs, 0.4 *mM* of each primer, and 2 unit Taq DNA polymerase (CinnaGen, Iran). The forward and reverse primers were:

Forward: 5′ GGCGTTGCCGCTCTGAATGC3′
Reverse: 5′ GAGGGACT GAGCTGGAC AACCAC3′

The PCR was done with initial denaturation at 94*°C* for 5 *min* and then the genome was amplified for 35 cycles. Each amplification cycle was performed at 94*°C* for 45 *s*, 60*^o^C* for 45 *s*, and 72*°C* for 45 *s*. The final extension was performed at 72*°C* for 5 *min*.

**Figure 1.**
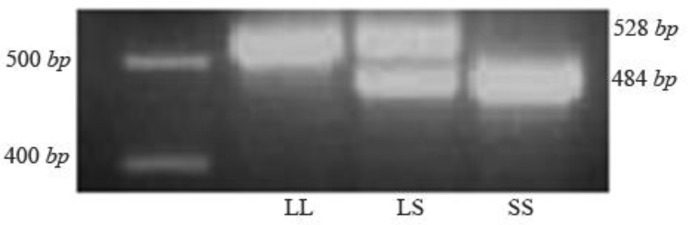
5-HTTLPR genetic polymorphism. Genotypes of 5-HTTLPR polymorphism determined by PCR method and analyzed by a 2.5% agarose gel electrophoresis stained with DNA safe stain and viewed under UV light. The size of the restriction fragments is shown.

PCR products were then analyzed by 2.5% agarose gel electrophoresis and visualized by DNA safe stain (CinnaGen, Iran). Individuals with SS genotype had a 484 *bp* fragment, those with LL genotype had a 528 *bp* fragment, and the individuals with (LS) genotype had two 484 *bp* and 528 *bp* bonds (Figure 1).

### Statistical analysis

Statistical analysis of the data was accomplished using SPSS software version 11.5 (SPSS Inc, Chicago, USA). A p-value of less than 0.05 was considered statistically significant (p≤0.05). Descriptive data were presented as mean±standard deviation (Mean±SD) and the qualitative variables were shown based on percentage. Chi-square test was used to measure the deviation from Hardy-Weinberg equilibrium and compare the two groups regarding the frequency of the alleles and genotypes. Moreover, the OR (Odds ratio) and its 95% confidence interval were calculated using logistic regression analysis. Finally, student t-test was used to compare the quantitative variables between the control and the patient groups, while one way ANOVA was used to compare the quantitative variables between different genotypes.

## Results

The results of PCR method showed the genotypes of 5-HTTLPR polymorphism (Figure 1). The biochemical and clinical profiles of the diabetic and normal control groups are shown in table 1. The groups were age matched (p=0.111), and the frequency of male and female subjects was similar (p=1.000) in the two groups. The mean of FBG, TG, total cholesterol, LDL cholesterol and HbA_1C_ levels in the diabetic patients was significantly (p<0.001) higher than the control group. HDL cholesterol was significantly (p< 0.001) lower in diabetic than control group. Genotype frequencies were in Hardy-Weinberg equilibrium in both groups. The frequency of LL, LS, and SS genotypes was respectively 46.7, 40, and 13.3% in the diabetic patients and 42.2, 44.4, and 13.3% in the normal subjects (Table 2). However, the difference regarding the frequency of genotypes and alleles was not statistically significant.

**Table 1.** Demographic and clinical variables of diabetic and normal control subjects

	**Control group (n=180)**	**Diabetic group (n=180)**	**p-value**
**Sex (M/F)**	84(46.7%)/96(53.3)	84(46.7%/96(53.3%)	1.000
**Age (years)**	62.24±7.51	59.89±6.34	0.111
**SBP (*mm Hg*)**	132.89±11.08	149.60±14.22	<0.001 ^*^
**DBP(*mm Hg*)**	86.70±7.29	89.74±7.14	0.049 ^*^
**BMI (*kg/m*^2^)**	26.29±3.21	29.01±2.27	0.001 ^*^
**HbA1C(%)**	5.40±0.64	7.55±1.18	<0.001 ^*^
**FBS(*mg/dl*)**	88.67±11.47	237.20±59.30	<0.001 ^*^
**TG (*mg/dl*)**	172.43±56.49	251.53±70.64	<0.001 ^*^
**Chol(*mg/dl*)**	195.66±61.88	285.84±64.72	<0.001 ^*^
**HDL(*mg/dl*)**	67.42±10.74	52.55±15.33	<0.001 ^*^
**LDL(*mg/dl*)**	128.24±64.50	183.57±60.78	<0.001 ^*^

Data was presented as frequency (percentage) and compared with chi-square test for sex. Also was presented with Mean±SD and compared with t test for other variables. Values of p≤0.05 were considered significant (*). F: Female, M: male, SBP: Systolic Blood Pressure, DPB: Diastolic Blood Pressure, BMI: Body Mass Index, Hb: Hemoglobin, FBS: Fasting Blood Sugar, TG: Triglyceride, Chol: Cholesterol, HDL: High Density Lipoprotein, LDL: Low Density Lipoprotein.

**Table 2.** The frequency of 5-HTTLPR genotypes and alleles in diabetic and normal control groups

	**Control**		**Experiment**		**Risk or protective genotype/allele**	**p-value**	**OR (95% CI)**
	
	**N**	**%**	**N**	**%**			
**Genotype**							
LL	76	42.2%	84	46.7%			
LS	80	44.4%	72	40.0%		0.902	
SS	24	13.3%	24	13.3%			
**Dominant model**							
LL	76	42.2%	84	46.7%			
LS + SS	104	57.8%	96	53.3%	LL	0.671	1.197 (0.790 to 1.815)
**Additive model**							
LS	80	44.4%	72	40.0%			
LL + SS	100	55.6%	108	60.0%	LS	0.670	0.833 (548 to 1.267)
**Recessive model**							
SS	24	13.3%	24	13.3%			
LL + LS	156	86.7%	156	86.7%	SS	1.000	1.000 (0.297 to 3.372)
**Alleles**							
L	232	64.4%	240	66.7%	L	0.754	1.103 (0.597 to 2.041)
S	128	35.6%	120	33.3%	S	0.754	0.906 (0.490 to 1.676)

LL and SS: homozygous genotypes, LS: heterozygous genotype. L: long allele, S: short allele. Values of p≤0.05 were considered significant. OR (95% CI): Odd Ratio 95% confidence interval.

Also, no significant differences were found between the T2DM patients carrying S allele (LS+SS) and those without S (LL) (OR=1.197, 95% CI=0.790–1.815) and also between the patients carrying L allele (LS+LL) and those without L (SS) (OR=1.000, 95% CI=0.297–3.372). Moreover, no significant differences were observed between the male and female subjects regarding the frequency of genes and alleles.

Clinical and biochemical factors of T2DM subjects according to genotype showed that the mean levels of TG, total cholesterol and LDL cholesterol in the diabetic patients carrying the L allele (LL+LS) were significantly higher than subjects without L allele (SS) (p<0.001). Also, Systolic Blood Pressure (SBP) was significantly higher in patients with L (LL+LS) allele compared to subjects without L allele (SS) (p=0.013). However, the mean levels of other profiles, including FBG and HbA_1C_, were not significantly different between the diabetic patients of different genotypes. The results of BMI showed that individuals who are not carriers for the long allele (SS) are known to be at risk for higher levels of obesity (p=0.029) (Table 3).

**Table 3.** Distribution of biochemical and clinical variables according to 5-HTTLPR genotypes in diabetic patients

	**Genotype**		

	**SS**	**LS+LL**	**p-value**
**Age (years)**	61.00±7.38	59.72±6.26	0.650
**SBP(*mm Hg*)**	136.38±6.51	151.63±14.02	0.013 ^*^
**DBP(*mm Hg*)**	85.13±3.11	90.45±7.34	0.090
**BMI (*kg/m*^2^)**	30.83±0.96	28.73±2.24	0.029 ^*^
**HbA1C(%)**	7.62±1.30	7.54±1.18	0.882
**FBS (*mg/dl*)**	233.88±61.80	237.71±59.73	0.885
**TG (*mg/dl*)**	148.85±36.09	267.32±60.72	<0.001^*^
**Chol (*mg/dl*)**	190.52±20.74	300.51±56.01	<0.001 ^*^
**HDL (*mg/dl*)**	57.88±9.64	51.73±15.96	0.366
**LDL (*mg/dl*)**	103.26±23.57	195.93±55.04	<0.001 ^*^

Data present as Mean±standard deviation and compared with t-test. Values of p≤0.05 were considered significant (which are bold*). SBP: Systolic Blood Pressure, DPB: Diastolic Blood Pressure, BMI: Body Mass Index, Hb: Hemoglobin, FBS: Fasting Blood Sugar, TG: Triglyceride, Chol: Cholesterol, HDL: High Density Lipoprotein, LDL: Low Density Lipoprotein.

## Discussion

Some studies were carried out in order to find the relationship between polymorphism of 5-HTTLPR and T2DM in different populations 6,17–19. The results of these studies were not the same in various habitats 6,17. In this study, the distribution and allele frequencies of 5-HTTLPR polymorphism and T2DM, obesity and serum biochemical profiles were investigated in Iranian population. It seems that these results are due to the differences in race, weather, hemisphere and nutrition conditions which are effective in genetic factors.

Iordanidou *et al* showed that in Greek population, S allele is a risk factor for T2DM which was independent of age and sex 6. On the other hand, Peralta-Leal *et al* indicated that there was no statistical association between 5-HTTLPR polymorphism and the development of T2DM in the Mexican population 8. Also, Hameed *et al* recently showed that there was no statistical association between 5-HTTLPR polymorphism and the development of T2DM in Pakistani population 18. The findings of our study revealed no relationship between 5-HTTLPR polymorphism and T2DM in the Iranian population and no significant difference was observed between the diabetic and healthy subjects regarding the frequency of genotypes and alleles. The difference between the results of our study and the one conducted by Iordanidou *et al* confirms the difference in the alleles' frequency in various populations 6. In our study, the S allele frequency was reported as 35% which is less than that of European and North American population (43%), South East Asian population (80%) and Pakistan population (61%) 22–25, and in agreement with another study on Iranian population 19.

On the other hand, in Pakistani population, although the frequency of S allele (61%) was different in comparison with the results of our study (33.3%), there was no relationship between T2DM and 5-HTTLPR polymorphism 18. In general, food diet, physical activity, and eating habits are effective in the prevalence of insulin resistance and incidence of type 2 diabetes. The difference between the two populations regarding the above-mentioned factors could also be another reason for the difference between the results of these studies 26.

Recently, Mohammadi *et al* reported the frequency of S allele (52.2%) in Kurdish population from western Iran 27 which is different from our study in control groups (35.6%). This difference may be due to the difference in specific diet, pure race and continental condition in west population of Iran.

Nazam *et al* in 2010 reported that the S allele related to serum TG increased in the diabetic patients but not cholesterol in population in south of Iran 19, but in this study, more serum biochemical profiles in larger population of Iran were investigated and the findings of this study showed that the diabetic subjects with L allele had higher levels of TG, total cholesterol and LDL in their serum. In the previous study, the authors mentioned that it is necessary to conduct studies with larger population 19. Overall, the present study showed no relationship between 5-HTTLPR polymorphism and T2DM; however, a relationship was found between L-allele and high levels of TG, cholesterol and LDL in the diabetic patients. Comings *et al* found that cholesterol levels were significantly greater in the LS heterozygotes than either LL or SS homozygotes in patients with heart disease. They mentioned that there was a significant association between LS heterozygosity and heart disease, angina, and heart attacks ^28^. So, it seems that the patients with T2DM carrying the L allele could be at high risk of heart disease.

Also, a direct association was observed between the presence of S allele and the increase in BMI in diabetic patients. These results are in agreement with the reports of Sookoian *et al* among adolescent and adult men from Argentina 5,20.

## Conclusion

This study completed and confirmed the previous work which was performed among the population in the south of Iran 19. The findings of the present study showed no relationship between the polymorphism of 5-HTTLPR L/S and T2DM in Iranian population. Nevertheless, the L allele was related to the increased serum lipids and blood pressure in the diabetic patients. So L allele could be a risk factor for heart disease in diabetic patients.

There were several limitations in this study. The first limitation is that most of the present population was selected from Iranians who were referred to Tehran for treatment and maybe new condition affected their serum profiles. Second, other genetic and environmental factors were ignored in the present study. On the other hand, maybe the number of subjects was not large enough in the current study and the lack of any association between 5-HTTLPR polymorphism and T2DM can be attributed to limited number of subjects.
